# The long non-coding RNA, GAS5, enhances gefitinib-induced cell death in innate EGFR tyrosine kinase inhibitor-resistant lung adenocarcinoma cells with wide-type EGFR via downregulation of the IGF-1R expression

**DOI:** 10.1186/s13045-015-0140-6

**Published:** 2015-04-29

**Authors:** Siyuan Dong, Xiaohan Qu, Wenya Li, Xinwen Zhong, Peiwen Li, Shize Yang, Xitao Chen, Mingrui Shao, Lin Zhang

**Affiliations:** Department of Thoracic Surgery, First Hospital of China Medical University, Shenyang, Liaoning Province 110001 People’s Republic of China

**Keywords:** NSCLC, Gefitinib resistance, GAS5, IGF-1R

## Abstract

**Background:**

Epidermal growth factor receptor-tyrosine kinase inhibitors (EGFR-TKIs) are approved for patients with recurrent non-small cell lung cancer (NSCLC). However, the efficacy of EGFR-TKIs in NSCLC therapy is limited by primary and acquired resistance. Recent studies have revealed that long non-coding RNAs (LncRNA) may be involved in EGFR-TKI resistance. Therefore, a better understanding of the interactive mechanisms underlying LncRNA-mediated EGFR-TKIs resistance may help us to improve clinical response rates.

**Method:**

To investigate the expression of growth arrest-specific 5 (GAS5) in lung adenocarcinoma, we performed real-time reverse-transcriptase polymerase chain reaction. The correlation between GAS5 expression levels and the samples’ clinicopathological features was also analyzed. Primary resistance to EGFR-TKIs was identified in the human lung adenocarcinoma cell line A549. Plasmid vectors were used to overexpress GAS5 in A549 cells. MTT (3-(4,5-dimethylthiazol-2-Yl)-2,5-diphenyltetrazolium bromide) colony formation assays and EdU (5-ethynyl-2’-deoxyuridine) assays were used to assess cell proliferation, and flow-cytometric analysis was used to evaluate the apoptosis rate. The expression levels of our target proteins, namely, EGFR, p-EGFR, ERK, p-ERK, Akt, p-Akt, IGF-1R (insulin-like growth factor 1 receptor), and p-IGF-1R, were analyzed by western blotting. A549 cells transfected with pcDNA-GAS5 were injected into nude mice. The transplanted mice were treated with gefitinib to study the effect of GAS5 on the resistance to EGFR-TKIs *in vivo*.

**Results:**

Our results showed that GAS5 was significantly downregulated in lung adenocarcinoma tissues compared with the paired adjacent non-tumorous tissue samples. Furthermore, lower GAS5 expression levels were associated with larger tumor sizes, poor tumor differentiation, and advanced pathological stages. However, GAS5 was almost equally expressed between benign tumors compared with the adjacent normal tissues. GAS5 was also overexpressed in EGFR-TKI sensitive cell lines compared with the resistant cell line. Using MTT, EdU incorporation, and colony formation assays, we showed that GAS5-expressing A549 cells displayed an elevated level of cell death. In addition to its pro-apoptotic effect in the A549 cell line, GAS5 overexpression also suppressed the growth of A549-derived tumors in nude mice treated with gefitinib. GAS5 overexpression was inversely correlated with the expression of the EGFR pathway and IGF-1R proteins.

**Conclusions:**

Collectively, our results indicated that GAS5 LncRNA may represent a potential biomarker for the diagnosis of lung adenocarcinoma and that GAS5 might play a novel role in the development of the resistance to gefitinib, which could be reversed by overexpressing GAS5.

## Introduction

Lung cancer continues to be one of the leading causes of cancer-related mortality worldwide accounting for 15% of all cancer diagnoses, and its incidence is increasing [1]. Lung adenocarcinoma is the major subtype of non-small cell lung cancer and is usually diagnosed at an advanced stage; therefore, patients miss the opportunity to receive surgery, which remains among the best treatments to date [2]. However, the promising benefits of epidermal growth factor receptor-tyrosine kinase inhibitors (EGFR-TKIs) have been clearly observed [3,4]. EGFR-TKIs are considered to be the standard first-line treatment for patients with advanced NSCLC harboring activating EGFR mutations [5,6]. However, the presence of intrinsic resistance is an obstacle for lung cancer treatment. Moreover, despite an initial dramatic response to inhibitors, drug resistance to the EGFR-TKI therapy almost always develops after approximately less than 1 year from the initiation of the treatment, which largely reduces the efficacy of these drugs [7]. Therefore, a better understanding of the molecular mechanisms underlying lung adenocarcinoma cell proliferation and apoptosis, as well as their relationship with EGFR-TKIs, is needed for improving the treatment of lung adenocarcinoma more efficiently.

In the past, investigations into the mechanisms of tumorigenesis mainly focused on protein-coding genes. Recently, a large number of non-protein-coding transcripts have been discovered. Although once thought to be a part of the genomic ‘dark matter’, more attention is now focused on long non-coding RNAs (LncRNAs). They are distinguished from short RNAs by their length, which is greater than 200 nucleotides, and have been shown to regulate many key biological processes [8]. Ewan found several LncRNAs with low expression in normal tissues but with high expression in both embryonic stem and cancer cells [9]. Beyond the expression changes, accumulating evidence indicates that the aberrant expression of some LncRNAs may play an important functional role in cancer biology [10,11]. Recent studies have also revealed that LncRNAs can act as proto-oncogenes (e.g., HOTAIR) or tumor suppressor genes (e.g., GAS5 (growth arrest-specific transcript 5)) in tumorigenesis [12,13].

GAS5 is a well-known LncRNA (−650 bases in humans and encoded at 1q25) that comprises 12 exons and encodes 10 box C/D snoRNAs within its introns. GAS5 was originally isolated from NIH 3 T3 cells and is receiving an increasing level of attention [14,15]. Several studies found that GAS5 sensitizes cells to apoptosis by regulating the activity of glucocorticoids in response to nutrient starvation and induces growth arrest and apoptosis independently of other stimuli in some breast cancer cell lines. In addition, the GAS5 transcript levels were found to be significantly reduced in breast and lung cancer samples compared with adjacent unaffected normal tissues [12,16].

However, the biological role of GAS5 and its function in EGFR-TKI-resistant lung adenocarcinoma remain largely unknown. In the present study, we found that GAS5 expression was significantly downregulated in lung adenocarcinoma tissues compared to the adjacent normal lung tissue, which is of clinical significance. Our results also showed that GAS5 overexpression reversed the gefitinib resistance of the human lung adenocarcinoma A549 cell line. Our work suggests that GAS5 LncRNA expression may represent a valuable marker for diagnosis and outcome predictions in patients with lung adenocarcinoma and that increased GAS5 expression may overcome the resistance to EGFR-TKIs in resistant lung adenocarcinoma, both *in vitro* and *in vivo*. Taken together, our results indicate that GAS5 plays an important role in lung adenocarcinoma development and could be a potential therapeutic target for lung adenocarcinoma patients.

## Results

### GAS5 is downregulated in lung adenocarcinoma tissues

**Figure 1:**
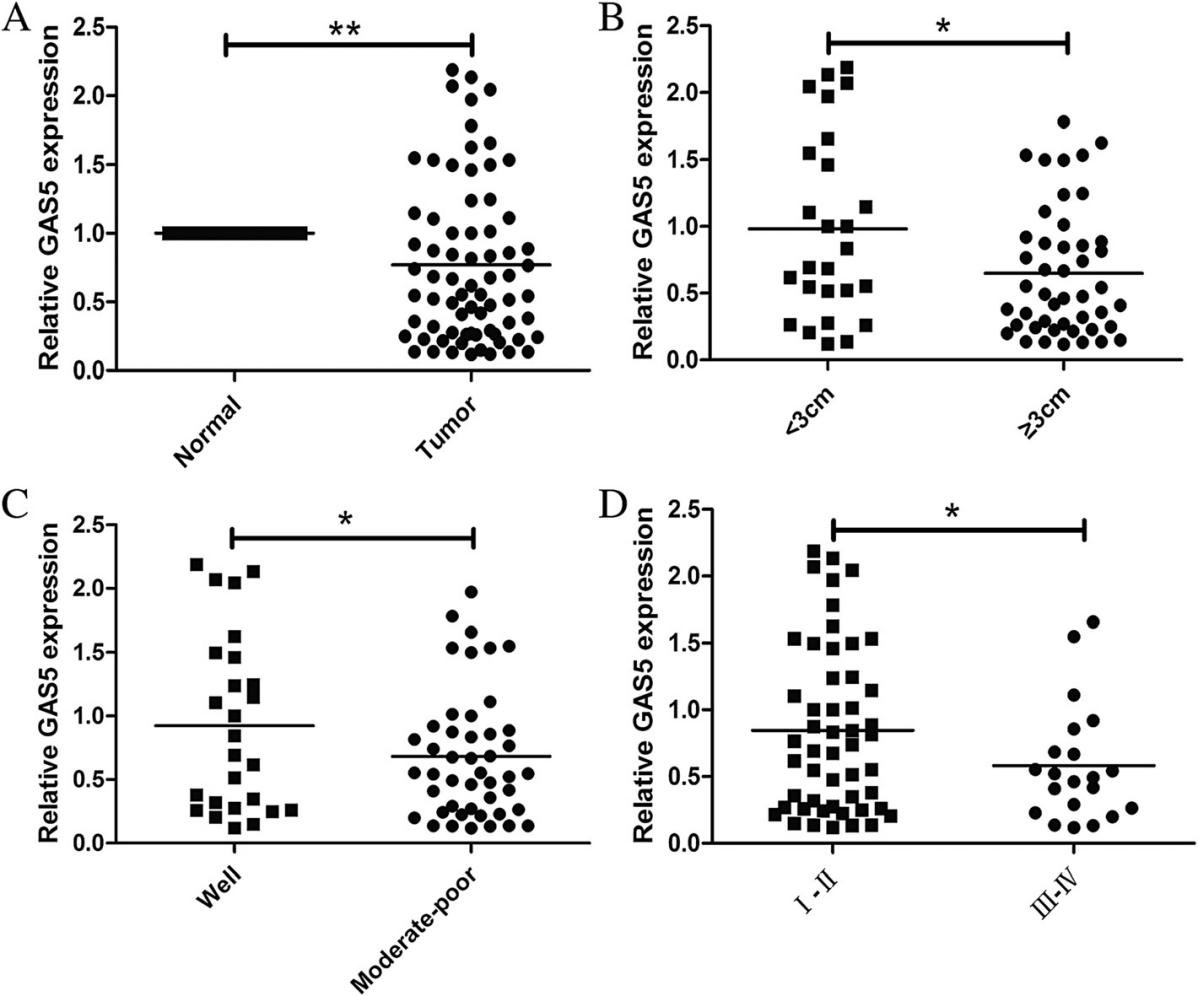
qRT-PCR analysis of LncRNA GAS5 in NSCLC tissues and its clinical significance. GAS5 expression was normalized to GAPDH expression. **(A)** GAS5 was measured in 72 pair NSCLC and paired adjacent normal tissues by qRT-PCR. The data are presented as a fold-change in the tumor tissue relative to the normal tissue. **(B)** GAS5 expression was significantly lower in the tumors ≥3 cm. **(C)** GAS5 expression was significantly lower in moderate-poor differentiation tumors. **(D)** GAS5 expression was significantly lower in patients with a higher pathological stage. *P < 0.05; **P < 0.01.

The quantitative reverse-transcriptase polymerase chain reaction (qRT-PCR) technique was used to assess GAS5 expression in tumor samples and their corresponding adjacent normal tissues in a total of 99 paired samples. In cancerous tissues, GAS5 expression was lower than the average level observed in normal specimens, with an average expression level of 0.76 compared with normal tissue (Figure 1A). In eight metastatic lung cancer tissues, GAS5 expression was also lower than that in the normal specimens, but not significantly (*P* = 0.087). In the 19 benign lung tumors, GAS5 expression levels were slightly lower than in the adjacent normal tissues, with an average expression level of 0.936 compared with normal tissues (*P* = 0.342).

The 72 NSCLC patients were classified into two groups: the relatively high-GAS5 group (*n* = 36, GAS5 expression ratio ≥ mean ratio) and the relatively low-GAS5 group (*n* = 50, GAS5 expression ratio ≤ median ratio). The examination of the correlation between the clinicopathological features and GAS5 expression levels showed that decreased GAS5 expression was correlated with a larger tumor size, poorer differentiation, and more advanced tumor-node-metastasis (TNM) staging (Figure 1B–D). However, there was no significant correlation between GAS5 expression and other factors, such as gender, age, smoking history, lymph node metastasis, distal metastasis, and carcinoembryonic antigen (CEA), carbohydrate antigen 19–9 (CA19-9), and cancer antigen 125 (CA125) expression (Table 1).

**Table 1:** **Clinicopathologic associations of GAS5 expression in lung adenocarcinoma**

**Clinical parameters**	**No. of cases**	**Relative GAS5 expression**		
		Low	High	*P* value^a^
Age				0.500
>60 years	39	19	20	
≤60 years	33	17	16	
Gender				0.479
Male	37	20	17	
Female	35	16	19	
Smoking				0.151
Non-smoker	42	18	24	
Smoker	30	18	12	
Differentiation				0.03**
Well	26	14	12	
Moderate-Poor	46	36	10	
Maximum diameter				0.03**
<3 cm	26	14	12	
≥3 cm	46	36	10	
Lymphatic metastasis				0.131
Absent	41	27	14	
Present	31	25	6	
Distal metastasis				1.000
M0	68	34	34	
M1	4	2	2	
TNM stage				0.047**
I–II	51	32	19	
III–IV	21	18	3	
CEA				0.583
Positive	34	16	18	
Negative	38	18	20	
CA19-9				0.500
Positive	39	19	20	
Negative	33	17	16	
CA125				0.577
Positive	29	16	13	
Negative	43	29	24	

^a^Chi-squared test. **P < 0.05.

### GAS5 expression and IC_50_ in four lung adenocarcinoma cell lines and the characteristics of the cell lines

**Figure 2:**
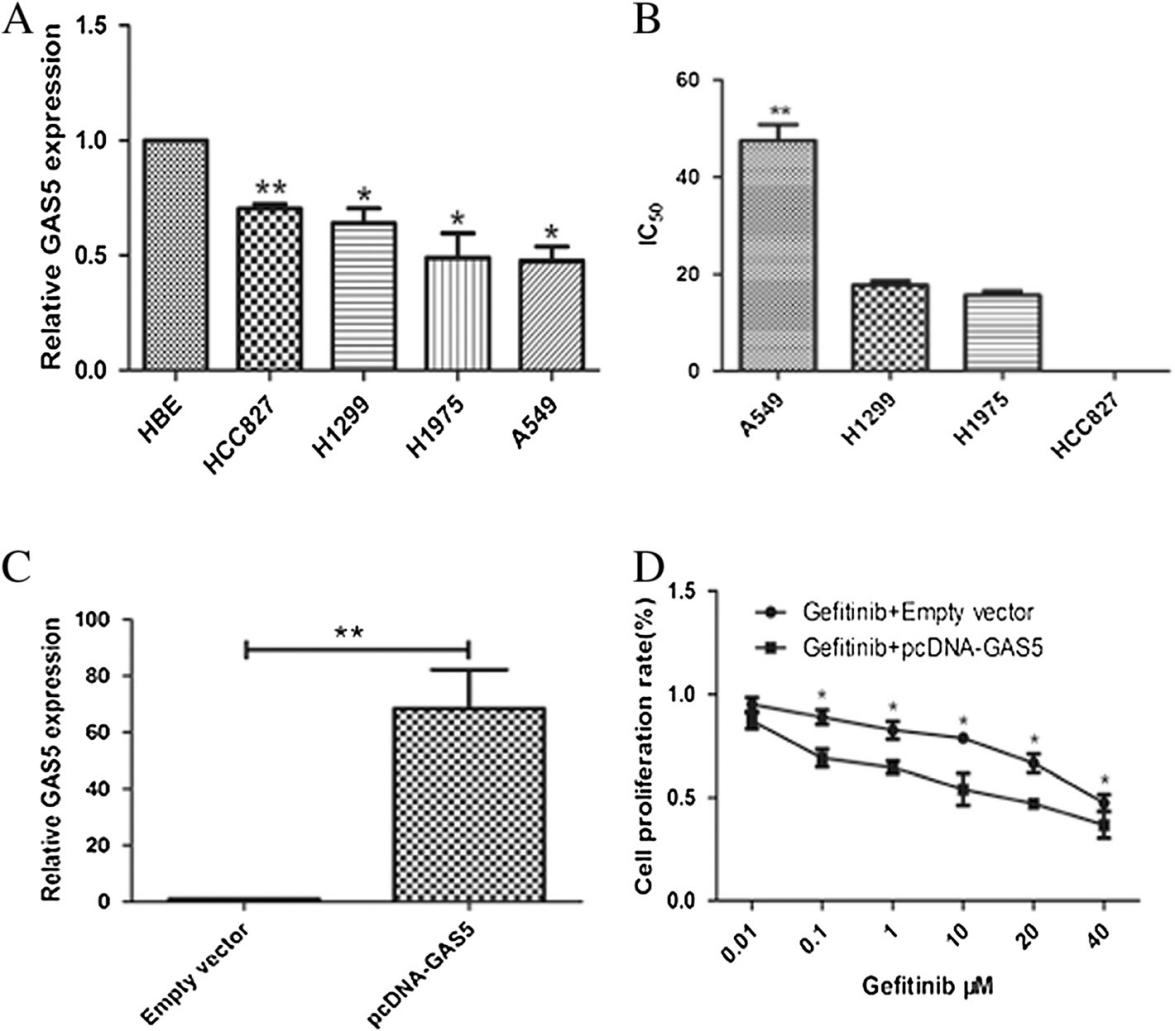
The GAS5 expression in lung adenocarcinoma cell line and the effect of gefitinib combined with GAS5 on the proliferation of A549 cell line **(A)** qRT-PCR results demonstrating GAS5 expression in NSCLC cell lines (A549, H1650, H1975, HCC827) compared to human bronchial epithelial cells (HBEs). **(B)** IC_50_ of four NSCLC cell lines. **(C)** GAS5 expression, measured by qRT-PCR, following the treatment of A549 cell line with pCDNA-GAS5 or empty vector. **(D)** The effect of GAS5 plus gefitinib on A549 proliferation *in vitro*. MTT assay was performed to determine the proliferation of pCDNA-GAS5- or empty vector-transfected A549 cells plus gefitinib.

To screen for the expression of GAS5 in the NSCLC cell lines, we used qRT-PCR analysis to assess GAS5 expression in the A549 (K-RAS mutant, EGFR wild-type), H1299 (EGFR wild-type, N-RAS), H1975 (T790M EGFR mutant), and HCC827 (EGFR mutant) lung adenocarcinoma cell lines. We found that GAS5 was expressed at a comparatively low level in the four lung adenocarcinoma cell lines compared with the human bronchial epithelial (HBE) cell line (Figure 2A). We defined the cell lines with a half maximal inhibitory concentration (IC_50_) value higher than 4 μM as gefitinib-resistant [17]. The IC_50_ of the A549, H1299, H1975, and HCC827 cell lines were 47.53 ± 3.30 μM, 17.72 ± 0.95 μM, 15.68 ± 0.89 μM, and 0.01 ± 0.001 μM, respectively (Figure 2B). As expected, the A549 was included in our study as the representative cell line for innate EGFR-TKI resistance.

### GAS5 treatment enhances gefitinib sensitivity *in vitro*

Because of the significance of LncRNAs in biological processes, we examined the impact of GAS5 overexpression on gefitinib resistance in the resistant lung adenocarcinoma cell line. GAS5 expression was significant upregulated 48 h after transfection, and qRT-PCR analysis showed that GAS5 expression was increased by 68-fold in A549 cells compared with the control cell line (Figure 2C).

An MTT (3-(4, 5-dimethylthiazol-2-yl)-2, 5-diphenyltetrazolium bromide) assay was used to measure the viability of the cells and their IC_50_ during treatments with increasing concentrations of gefitinib for 72 h. Compared with gefitinib treatment alone, all of the cells transfected with GAS5 exhibited a significantly decreased viability (Figure 2D), and the IC_50_ of the A549 cell line was decreased to 3.665 μM (Table 2).

**Table 2:** **GAS5 increases the sensitivity of A549 to gefitinib**

**Cell line**	**IC**_**50**_	**IC**_**50**_	**Ratio**
	**Gefitinib**	**Gefitinib + GAS5**	
A549	46.40 μM	3.665 μM	12.66

**Figure 3:**
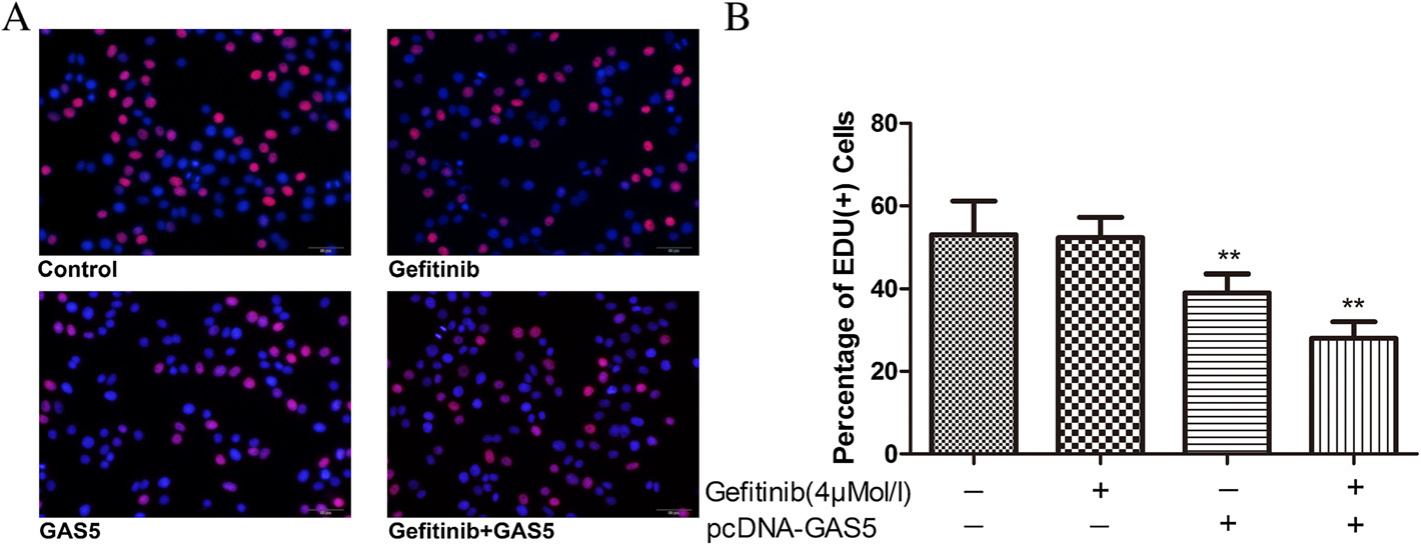
Effects of GAS5 plus gefitinib on cell proliferation in gefitinib-resistant A549 cells. **(A)** Representative EdU results of four groups. **(B)** Effects of GAS5 plus gefitinib on EdU+ stained cells at 6 h after treatment. The data represent the mean ± SD from three independent experiments. **P < 0.01

An EdU (5-ethynyl-2’-deoxyuridine) incorporation assay was used to detect DNA synthesis, which is an indicator of cell proliferation. As show in Figure 3, the percentage of EdU-positive cells in the S phase of the cell cycle was significantly lower in the combination group than in the control and gefitinib alone group (*P* < 0.05).

**Figure 4:**
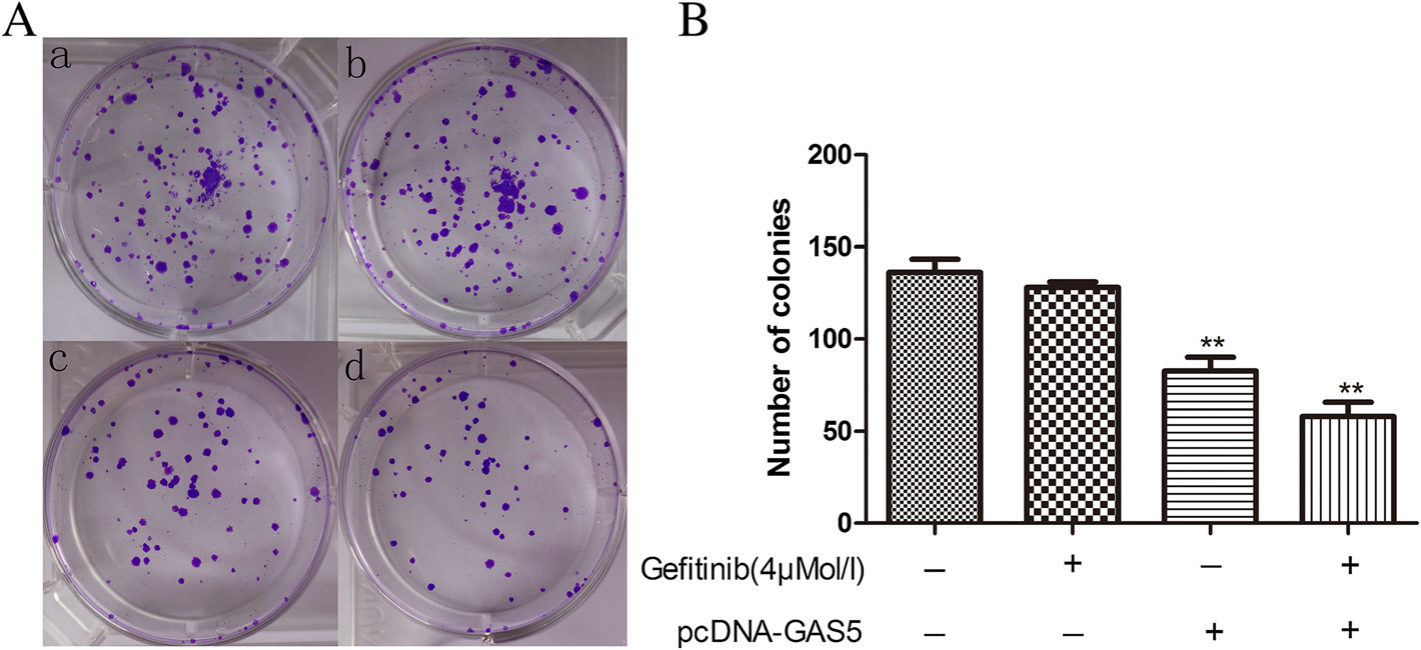
A colony-forming growth assay was performed to determine the proliferation of pCDNA-GAS5- or empty vector-transfected A549 cells plus gefitinib. The colonies were counted and captured. **(A)** Representative colony formation assay results of four groups. **(B)** GAS5 and gefitinib together synergistically suppress colony formation. The data represent the mean ± SD from three independent experiments. **P < 0.01

In the colony formation assay, we obtained similar results. The clonogenic survival was greatly decreased following GAS5 overexpression in A549 cells (Figure 4).

**Figure 5:**
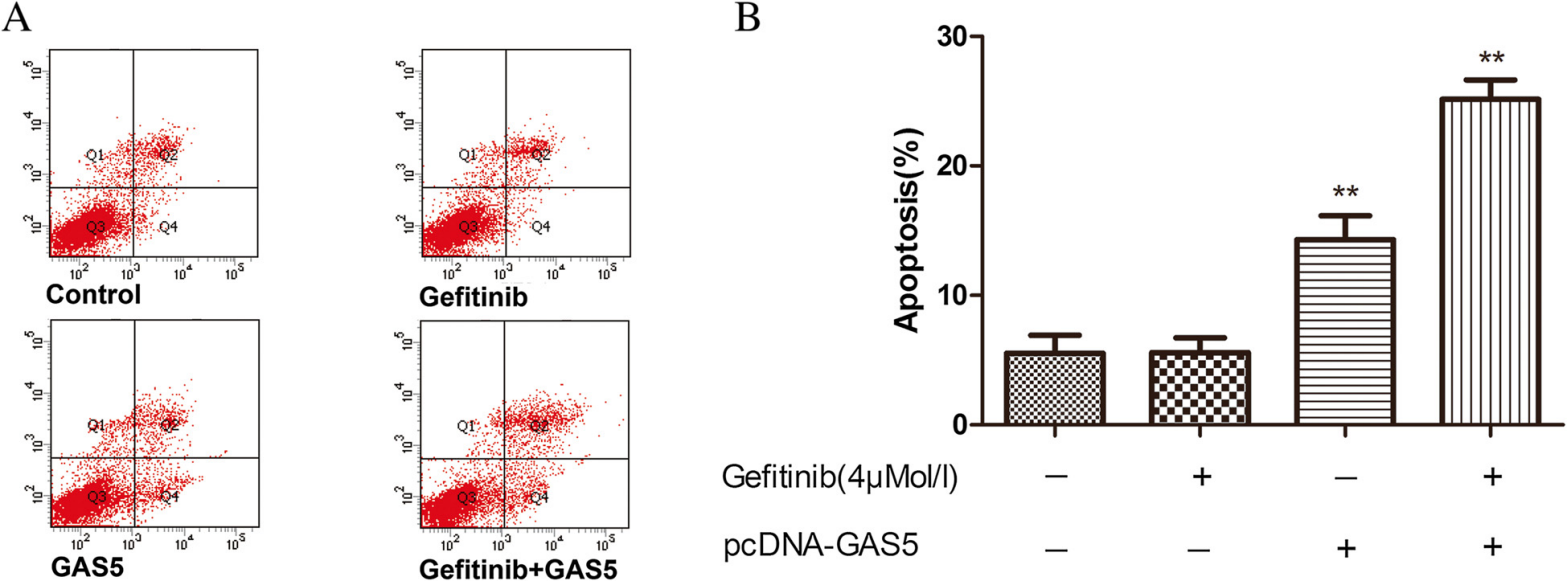
Flow-cytometric analysis was used to determine the percentage of apoptotic cells. **(A)** Representative flow-cytometric analysis results of four groups. **(B)** A549 cell treated with gefitinib plus GAS5 showed decreased apoptosis. The data represent the mean ± SD from three independent experiments. **P < 0.01

Flow cytometry was used to analyze apoptosis in A549 cells exposed to gefitinib for 72 h at a concentration of 4 μM. The cells transfected with GAS5 showed a significantly higher apoptosis rate compared with the control cells (Figure 5).

Collectively, these data revealed that GAS5 could sensitize the resistant A549 cell line to the cytotoxic effects of gefitinib.

### GAS5 regulates the sensitivity to EGFR-TKIs by inhibiting IGF-1R

**Figure 6:**
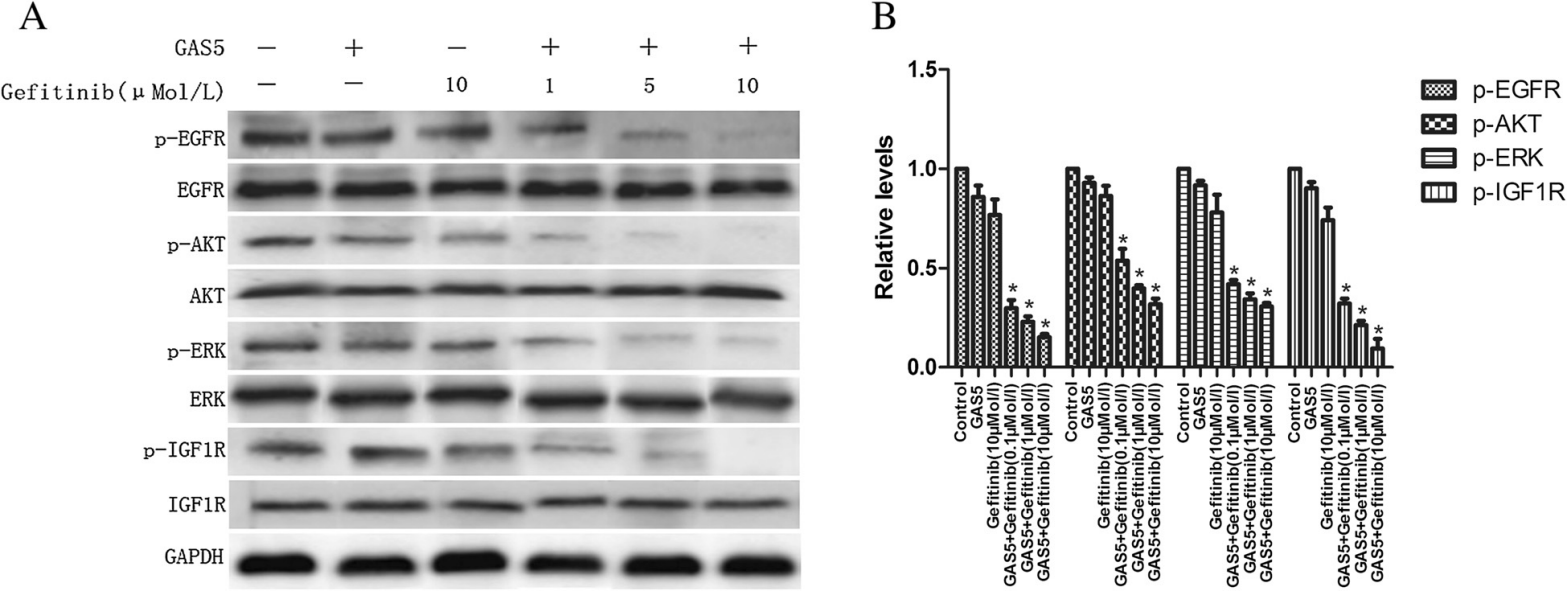
Effect of GAS5 and gefitinib on the activity of the EGFR/PI3K/AKT pathway and IGF-1R in A549 cells. **(A)** Significant suppression of EGFR and downstream signaling molecules are seen following co-treatment with GAS5 and gefitinib. Dose-dependent downregulation of IGF-1R protein was also seen. **(B)** Relative levels of P-EGFR, P-AKT, P-ERK and P-IGF-1R proteins expression in six groups. The data represent the mean ± SD from three independent experiments. *P < 0.05

Western blotting analyses were conducted to evaluate the mechanism behind the enhanced effect of the combined treatment. We assessed the protein expression levels of EGFR and the activation of its downstream signaling proteins AKT and ERK. In A549 cells, gefitinib or GAS5 alone minimally inhibited the protein levels of phospho-EGFR, phospho-ERK, and phospho-Akt. Furthermore, we observed a decrease in the expression of phospho-EGFR and the downstream signaling proteins after the combination treatment (gefitinib and GAS5 overexpression) compared with the monotherapy (*P* < 0.05). In addition, gefitinib combined with GAS5 decreased the levels of phospho-ERK and phospho-Akt in a dose-dependent manner in A549 cells (Figure 6).

The IGF-1R (insulin-like growth factor 1 receptor) protein level, which has been associated with resistance to EGFR-TKIs, was also analyzed to explore the potential mechanisms behind the effects of the GAS5/gefitinib combination on gefitinib-resistant lung adenocarcinoma cells. As shown in Figure 6, A549 cells treated with GAS5 plus gefitinib showed a significant reduction in the phospho-IGF-IR level. These results indicate that the tyrosine kinase receptors, which play important roles in pro-oncogenic signaling in lung adenocarcinoma, were inactivated and that the cytotoxicity of gefitinib was potentiated by the combined treatment.

### GAS5 treatment enhances gefitinib sensitivity *in vivo*

**Figure 7:**
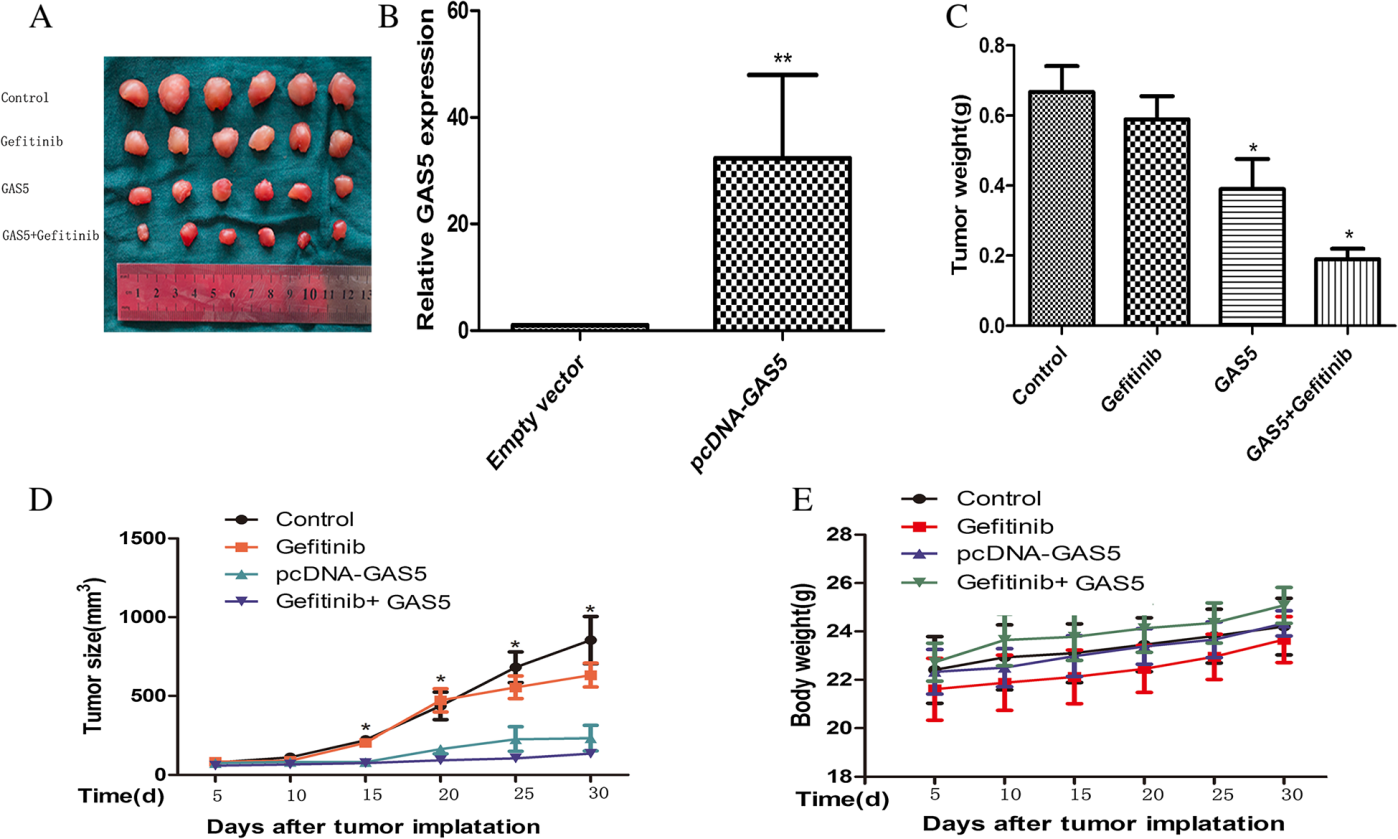
GAS5 in combination with gefitinib suppresses the growth of EGFR inhibitor-resistant tumor xenografts *in vivo*. **(A)** All the tumors after removal from the mice after 30 days. **(B)** qRT-PCR analysis of GAS5 expression in tumor tissues formed from four groups, and qRT-PCR analyses indicated that GAS5 expression is significantly increased *in vivo*. **(C)** Tumor weight when the tumors were harvested. The data represent the mean ± SD. **(D)** Tumor growth curve. Six mice per group were used in the xenograft experiment. The tumor volumes of mice were measured. *P < 0.05, for comparisons with gefitinib alone. **(E)** The gefitinib treatments did not cause significant body weight loss in the tested mice.

Although GAS5 can restore sensitivity to gefitinib in A549 cells, there is little direct evidence for the role of GAS5 *in vivo*. A xenograft mouse model was used to provide further evidence of the role of GAS5 in reversing gefitinib resistance *in vivo*. Five days after the injection of empty vector- or GAS5-expressing A549 cells, detectable tumors developed in both groups. The GAS5 overexpression treatment dramatically decreased the tumor size and weight compared with the control group and the gefitinib only group (Figure 7A, C, D). The qRT-PCR analysis was conducted to confirm the upregulation of GAS5 in tumors after pcDNA-GAS5 transfection (Figure 7B). In addition, we monitored the health and body weight of the mice, which indicated that the mice tolerated all of the treatments without displaying any overt signs of toxicity (Figure 7E).

**Figure 8:**
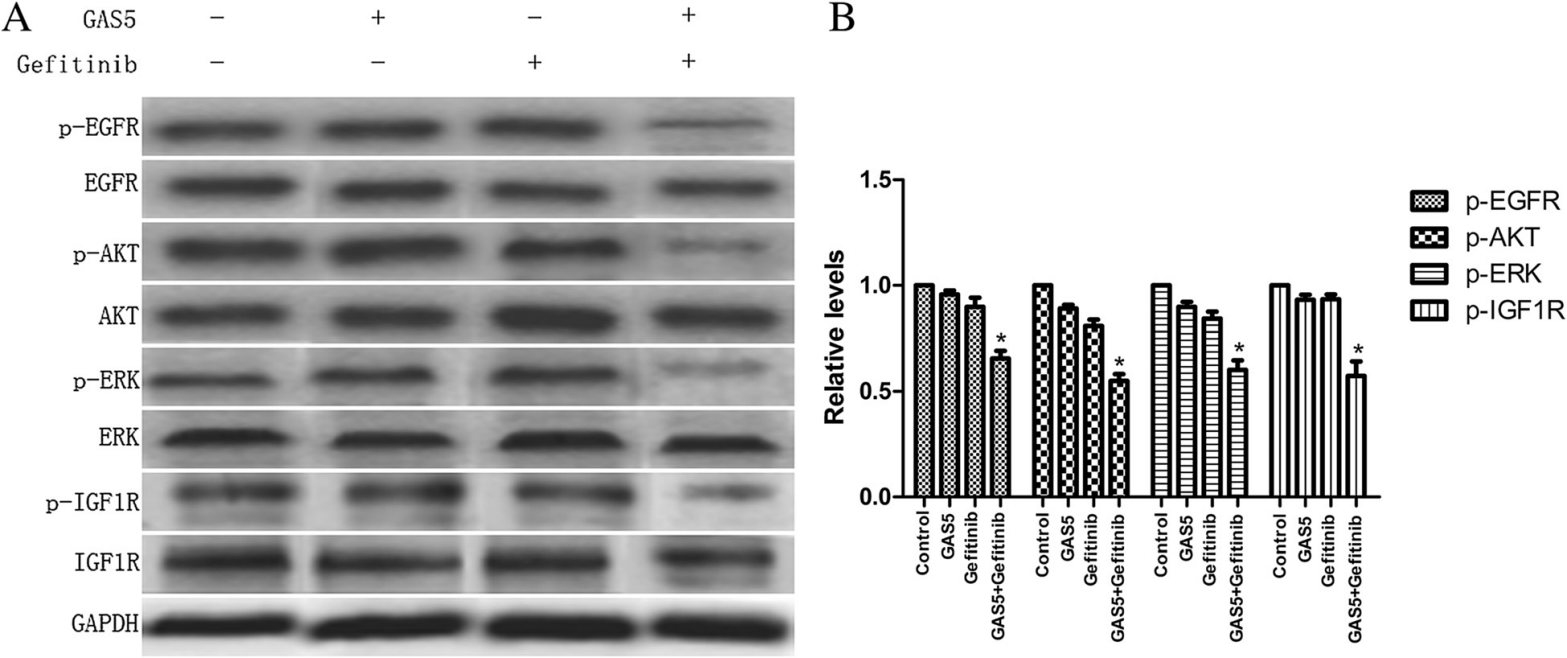
Effect of GAS5 and gefitinib on the activity of the EGFR/PI3K/AKT pathway and IGF-1R *in vivo*. **(A)** Significant suppression of EGFR, downstream signaling molecules, and IGF-1R are seen following co-treatment with GAS5 and gefitinib. **(B)** Relative levels of P-EGFR, P-AKT, P-ERK, and P-IGF-1R proteins expression in four groups. The data represent the mean ± SD from three independent experiments. *P < 0.05

The total proteins were isolated from the A549 xenograft tumors, and western blotting analyses were performed to detect the expression of EGFR and the activation of its downstream signaling proteins. Our results revealed that the combined treatment markedly reduced the levels of phospho-EGFR and the downstream signaling proteins phospho-AKT, phospho-ERK, and phospho-IGF-1R within the tumors (Figure 8). Our results revealed that GAS5 is a promising molecule that is capable of overcoming the resistance to EGFR inhibitors in lung adenocarcinoma.

## Discussion

EGFR is a transmembrane protein with an extracellular ligand-binding domain and an intracellular domain possessing an intrinsic tyrosine kinase activity. The overexpression of EGFR in NSCLC patients is always associated with poor prognosis [18]. EGFR-TKIs that target the tyrosine kinase activity of EGFR have been developed. However, the limited response rate is an obstacle to their use for the treatment of NSCLC. Therefore, overcoming the primary or secondary resistance to EGFR-TKIs remains one of the most significant challenges for the successful treatment of lung cancer. To solve this problem, new strategies have been developed against EGFR-TKI-resistant cells. However, the combination of EGFR-TKI treatment with chemotherapeutic agents caused a significant level of toxicity for the patients and only achieved a modest increase in survival [19,20]. Therefore, the identification of alternative approaches preventing the growth of EGFR-TKI-resistant NSCLC cells is of critical importance.

Understanding the molecular processes involved in NSCLC has led to new treatment options. Recently, the importance of the epigenetic changes that occur during cancer development was recognized. In total, only 2% of the human genome is associated with protein-coding genes and the majority of the human genome is associated with non-coding RNAs (ncRNAs). An increasing number of studies show that ncRNAs could play important regulatory roles in complex organisms [21]. As an important part of epigenetics, LncRNAs are gaining the attention of researchers in many fields, and the number of published articles related to their role in cancer is exponentially growing [22]. In the present study, our attention was focused on the GAS5 LncRNA. GAS5 is a tumor suppressor gene that is downregulated in various types of cancer [23,24]. The downregulation of GAS5 expression is thought to contribute to tumor formation and to affect proliferation and apoptosis [16].

We found that the GAS5 expression levels were significantly downregulated in lung adenocarcinoma lesions compared with the adjacent noncancerous tissues, which was consistent with previous findings in other cancer types [23,24]. Our current results also show a lower GAS5 expression in metastatic lung cancer tissues, although this difference was not statistically significant, possibly because of the limited number of cases analyzed. More cases of metastatic tissue samples may be needed to evaluate the relationship between low GAS5 expression and pulmonary metastases. Tumor size and differentiation status are two key biological features that determine the aggressiveness of lung adenocarcinoma tumors and represent the major obstacles to the treatment of these malignancies. The most significant factors affecting the prognosis of patients with lung adenocarcinoma are considered to be the tumor size and the degree of local invasion of the tumor. The potential relationship between GAS5 levels in tumors and the clinicopathological features of lung adenocarcinoma patients were investigated. We observed that low GAS5 expression was associated with a larger tumor size and a poorer differentiation status. Furthermore, our study is the first to elucidate the relationship between GAS5 and benign lung tumors. Indeed, we found that benign lung tumors display an almost equal GAS5 expression level as the normal surrounding tissues. This finding supports the hypothesis that GAS5 acts as a tumor suppressor gene in lung adenocarcinoma.

To solve the problem of EGFR-TKI resistance, several new strategies have been developed [17,25]. In addition, epigenetic therapy has also emerged as a new approach for cancer treatment. GAS5 can modulate cellular responses by suppressing the glucocorticoid-mediated induction of several response genes, inducing cell cycle arrest, inhibiting cell growth, and inducing apoptosis [26,27]. A recent study has revealed that erlotinib, a tyrosine kinase inhibitor that acts on the epidermal growth factor receptor, can significantly affect the expression of the GAS5 gene in glioma cells [28]. However, the connection between the GAS5 levels and the sensitivity to EGFR-TKIs in tumor cells is unclear and remains to be elucidated. Here, we explored the effects of modulating the levels of the GAS5 LncRNA in combination with gefitinib treatment using *in vitro* and *in vivo* models. We examined the cytotoxicity of a combination of gefitinib and GAS5 in a primary resistant cell line. A synergistic effect was consistently observed for a number of parameters, including apoptosis and cytotoxicity. Compared with gefitinib or GAS5 alone, all of the cells treated with gefitinib plus GAS5 exhibited a dose-dependent decrease in viability. Our results suggest that the gefitinib/GAS5 co-treatment may overcome primary resistance. However, because GAS5 is known to act as decoy for the glucocorticoid receptor, we will next investigate whether glucocorticoid signaling plays a role in the resistance to EGFR-TKIs and investigate which GR target genes could be related to the development of resistance.

EGFR-TKIs can inhibit the downstream effects of the EGFR pathway, resulting in an inhibition of cell proliferation, invasion, and survival. Our combined treatment downregulated EGFR and p-EGFR expression in the A549 cell line and also resulted in a reduction of both Akt and ERK phosphorylation. Our results suggest that GAS5 in combination with gefitinib inhibited EGFR activity and the phosphorylation of its downstream pathway components, which is important to overcome resistance [29,30], and, finally, restored the sensitivity of lung adenocarcinoma cell lines to the EGFR-TKIs.

Other studies have found that the overexpression of IGF-1R was associated with resistance to EGFR-TKI treatments [31,32]. The Guix’s group study revealed that treating the EGFR-TKI-resistant A431 cell line with an IGF-1R inhibitor restored their sensitivity [33]. The interplay between LncRNAs and proteins is a significant matter in the field of cancer biology. Previous studies have shown that the interaction between LncRNAs and IGF-1R was complicated. Indeed, Aparna et al. found that the maternal-specific H19-DMR deletion led to the upregulation of Igf2 and to an increase in IGF-1R translation, the latter of which is normally suppressed by H19-derived miR-675 [34]. Patients with squamous cell carcinoma overexpress IGF-1R more frequently than the patients with a nonsquamous histology [35]. Therefore, we hypothesized that GAS5 may also mediate IGF-1R function to enhance the sensitivity to EGFR-TKIs in lung adenocarcinoma. Our results demonstrated that GAS5 could directly downregulate IGF-1R expression and, as a result, decrease cell viability and resistance to EGFR-TKIs, which suggests that IGF-1R was also a downstream target of GAS5 in the resistance to EGFR-TKI. Zhang et al. [36] showed that miR-21 and GAS5 can regulate each other in a similar way as the microRNA-mediated silencing of target mRNAs. Because of the correlation between IGF-1R and Mir-21 [37], we speculate that GAS5 might downregulate IGF-1R by affecting Mir-21. In this study, we demonstrated, for the first time, that GAS5 exerts a tumor suppressive function by regulating IGF-1R expression in lung adenocarcinoma. However, more studies are needed to determine the pathway that is primarily responsible for the biological consequences of GAS5 overexpression.

To confirm the importance of GAS5 in gefitinib resistance, we examined whether the overexpression of LncRNA could affect the gefitinib-induced cytotoxicity *in vivo*. These transplanted cells showed a high propensity for invasive growth within the surrounding tissues. The combined treatment was satisfactory and enhanced cytotoxicity and tumor suppression. Next, we further validated our western blotting results *in vivo*. Our results revealed a reduction of the phospho-EGFR, phospho-Akt, phospho-ERK, and phospho-IGF-1R levels *in vivo*. Collectively, these results clearly indicate that GAS5 can overcome EGFR-TKI resistance by inhibiting IGF-1R *in vivo*.

In this work, we report, for the first time, that GAS5 downregulation is not only associated with adenocarcinoma tumorigenesis and progression but is also associated with primary resistance to EGFR-TKIs, both *in vitro* and *in vivo*. GAS5 may become a new tool in our repertoire of anticancer agents for the therapy of NSCLC. Furthermore, although we identified IGF-1R as a key downstream mediator of GAS5 in the resistance to EGFR-TKIs, the underlying molecular mechanisms and detailed signaling pathways regulating GAS5 remain to be elucidated. Because GAS5 could be easily detected in tissue samples, its potential role as an indicator of EGFR-TKIs’ efficacy and as a target to overcome resistance to EGFR-TKIs requires further in-depth investigation. Importantly, only wild-type EGFR-expressing cells were included in our study. Further studies are needed to explore whether this co-treatment may also overcome resistance in other contexts.

## Conclusion

Our study shows, for the first time, that the upregulation of GAS5 can overcome the resistance of human adenocarcinoma cells to EGFR-TKIs, at least partially by downregulating IGF-1R. Our results support a potential therapeutic role for a combinatorial epigenetic platform for the treatment of lung adenocarcinoma, particularly in patients with primary EGFR-TKI resistance. Collectively, our study explores the validity of using GAS5 as a new treatment approach in lung adenocarcinoma. In the future, the feasibility of this approach should be evaluated via clinical trials for patients with lung adenocarcinoma.

## Materials and methods

### Cell lines and cell culture

Human lung adenocarcinoma cell lines (A549, H1299, H1975, HCC827) and HBE cells were obtained from the Institute of Biochemistry and Cell Biology of the Chinese Academy of Sciences (Shanghai, China). The cells were cultured in RPMI 1640 medium containing 10% fetal bovine serum (FBS), 100 U/ml of streptomycin, and 2 mM of L-glutamine under a 5% CO_2_-containing humidified atmosphere at 37°C.

### Patients and tissue samples

Between February 2013 and June 2014, a total of 72 paired cancerous and matched adjacent noncancerous lung tissues were collected from lung adenocarcinoma patients undergoing lobectomy. These patients included 37 men and 35 women, ranging in age from 38 to 75 years, with a median age of 60 years. No patients received any preoperative chemotherapy or radiotherapy, and no patients had a previous history of malignant disease. Samples from 8 cases of metastatic lung cancer, 19 cases of benign lung tumor, and their corresponding normal lung tissues were also collected. The 8 metastatic lung cancer cases included 4 cases of colorectal metastatic lung cancer, 3 cases of breast metastatic lung cancer, and 1 case of renal cell metastatic lung cancer. The 19 cases of benign tumors included 8 cases of inflammatory pseudotumor, 6 cases of tuberculoma, 3 cases of pulmonary hamartoma, and 2 cases of atypical adenomatous hyperplasia (AAH). All of the tumor tissues were diagnosed histopathologically by at least two trained pathologists. The clinicopathological characteristics of the included patients with lung adenocarcinoma are summarized in Table 1, and the characteristics of the included patients with metastatic lung cancer and benign tumors are shown in Table 3.

**Table 3:** **GAS5 expression in metastatic lung cancer and benign lung tumor**

	**No. of cases**	**Relative GAS5 expression**		
		**Low**	**High**	***P*** value*
Metastatic lung cancer				0.272
Colorectal cancer metastases	4	3	1	
Breast cancer metastases	3	2	1	
Renal cell cancer metastases	1	1		
Benign lung tumor				0.823
Inflammatory pseudotumor	8	5	3	
Tuberculoma	6	3	3	
Pulmonary hamartoma	3	1	2	
Atypical adenomatous hyperplasia	2	1	1	

*Two-tailed Student’s *t*-test.

After surgery, the resected primary tumors and normal tissues were immediately frozen in liquid nitrogen and stored at −80°C until the RNA extraction was performed. The TNM classification and tumor staging were performed according to the classification criteria of the World Health Organization and the TNM stage criteria, as determined by the Union for International Cancer Control [38].

### RNA isolation and qRT-PCR analyses

Total RNA was isolated with TRIzol reagent (Invitrogen, Carlsbad, CA, USA) according to the manufacturer’s protocol. The isolated RNA was reverse-transcribed into cDNA using a reverse transcription kit (Takara, Dalian, China). The results were normalized against glyceraldehyde 3-phosphate dehydrogenase **(**GAPDH) expression. The PCR primers for GAS5 or GAPDH were as follows: GAS5 sense, 5’ CCCAAGGAAGGATGAG3’ and reverse, 5’ ACCAGGAGCAGAACCA3’; GAPDH sense, 5’-CAATGACCCCTTCATTGACC-3’ and reverse, 5’- TGGAAGATGGTGATGGGATT-3’. qRT-PCR data collection was performed on an ABI 7500 apparatus (Applied Biosystems, Foster City, CA, USA). The PCR reaction was conducted for 2 min at 50°C, 10 min at 95°C, 40 cycles at 95°C for 15 s, and 60°C for 30 s. The relative quantification of GAS5 expression was calculated using the 2^−DDCT^ method relative to GAPDH levels. All of the qRT-PCR reactions were performed in duplicate.

### Ethics statement

Written informed consent was obtained from all of the patients. The study was approved by the Ethics Committee of the First Hospital of the China Medical University and was performed in compliance with the Helsinki Declaration. All of the experiments involving animals were performed in strict accordance with the recommendations of the Guide for the Care and Use of Laboratory Animals of the National Institutes of Health.

### Construction of the plasmid vector and DNA transfection

Based on the GAS5 sequence available on National Center for Biotechnology Information database, the full-length GAS5 sequence lacking a poly-A tail was synthesized and subcloned into pcDNA3.1 (Jikai, Shanghai, China). The empty vector or the pcDNA-GAS5 vector was transfected into A549 cell cultured in six-well plates using Lipofectamine 2000 (Invitrogen), according to the manufacturer’s instructions. The empty pcDNA3.1 vector was used as the negative control. The total RNA was isolated using the TRIzol reagent (Takara, Dalian, China) 48 h after the transfection, and the expression level of GAS5 was detected by qRT-PCR.

### *In vitro* drug sensitivity and cell viability assay

Gefitinib (Iressa) was purchased from AstraZeneca (London, England). Cellular proliferation under treatment with various doses of gefitinib after transfection was quantified using an MTT assay. A549 cells were seeded in 96-well plates at a density of 5 × 10^3^ cells/well and incubated overnight at 37°C. The cells were exposed to serial dilutions of gefitinib (0.01 mM, 0.1 mM, 1 mM, 10 mM, 20 mM, and 40 mM) for 48 h at 37°C. Then, 15 μl of an MTT reagent (0.5 mg/ml in PBS, Sigma St. Louis, MO, USA) was added into each well. Four hours later, the medium was replaced with 200 μl of dimethyl sulfoxide (DMSO, Sigma, St. Louis, MO, USA). The optical density was measured at a wavelength of 570 nm using a spectrophotometer. The IC_50_ value was determined as the concentration at which each drug produced 50% inhibition of growth, which was estimated from the relative survival curves and calculated by using the GraphPad 5 software (Prism). All of the experiments were performed in triplicate.

### Colony formation assay

A549 cells were transfected with pcDNA-GAS5 or the empty vector and placed onto a fresh six-well plate at a density of 1 × 10^4^ per well. The cells were maintained in media containing 10% FBS and exposed to gefitinib at a concentration of 25 μg/ml for 24 h. Then, the drugs were washed away and the medium was changed every 3 days for 2 weeks. After 15 days, the colonies were fixed with methanol and stained with a 0.5% crystal violet solution (Sigma, St. Louis, MO, USA). The colonies that were larger than 200 μM were manually counted. The experiments were repeated at least twice.

### EdU incorporation assay

Newly synthesized DNA after the indicated treatment in A549 cells was detected by EdU fluorescence staining according to the manufacturer’s instructions (Click-iT® EdU Imaging Kits, Invitrogen). The cells, cultured in a well of a 24-well plate at a cell density of 30,000 cells per well, were labeled with 10 μM of EdU and incubated for an additional 2 h before being fixed with 3.7% formaldehyde for 15 min at room temperature. The fixative was removed and the cells in each well were washed twice with 1 ml of 3% BSA in PBS. The BSA was removed and 1 ml of 0.5% Triton® X-100 (Sigma, San Francisco, CA ,USA) in PBS was added to each well and incubated at room temperature for 20 min. After washing the cells in each well twice with 3% BSA in PBS, the cells in each well were reacted with 500 μL of 1× Click-iT® reaction cocktail for 30 min at room temperature in the dark. Subsequently, for nuclear staining, 1 ml of 1× Hoechst 33342 solution (Sigma, San Francisco, CA, USA) was added to each well and incubated for 30 min at room temperature in the dark. The Hoechst 33342 solution was removed and the EdU-labeled cells were counted using fluorescence microscopy (CKX41-F32FL, Olympus, Tokyo, Japan) and normalized to the total number of Hoechst-stained cells. The Image-Pro Plus software (Version 6.0, Media Cybernetics, Bethesda, MD, USA) was used to calculate the percentage of EdU-positive (EdU+) cells.

### Flow cytometry analysis of apoptosis

The pcDNA-GAS5- or empty vector-transfected A549 cells were treated with 4 μM of gefitinib and cultured in six-well plates for 72 h. Then, the cells were harvested by trypsinization, and apoptosis was evaluated using an Annexin V-PE/7-AAD Apoptosis Detection Kit (BioVision, MA, USA). After the double staining with Annexin V-PE and 7AAD, flow cytometry analysis (FACScan, BD Biosciences, San Jose, CA, USA) using the CellQuest software (BD Biosciences) was used to quantify apoptosis. The cells were classified into dead cells (Q1), early apoptotic cells (Q2), viable cells (Q3), and late apoptotic cells (Q4).

### Western blotting

The total cellular and tissue protein extracts were separated on 10% SDS-polyacrylamide gels (SDS-PAGE) and transferred onto nitrocellulose membranes (Millipore, Bedford, MA, USA). The membranes were blocked and incubated with primary antibodies overnight at 4°C. A Bio-Rad protein assay kit was used to determine the protein concentration. The specific antibodies for p-EGFR, EGFR, p-AKT, AKT, p-ERK, ERK, and IGF-1R were purchased from Santa Cruz Biotechnology (Santa Cruz, CA, USA), and the antibodies for p-IGF-1R and anti-GAPDH were obtained from Cell Signaling Technology (Beverly, MA, USA). GAPDH was used as a control. The membranes were incubated with the appropriate horseradish peroxidase-conjugated monoclonal secondary antibody (Santa Cruz) at room temperature for 1.5 h. An ECL detection kit (ThermolBiotech, Boston, MA, USA) was used to visualize the immunoreactive protein bands. Densitometry (Quantity One Software; Bio-Rad, Hercules, CA, USA) analysis was used to quantify the relative levels of protein expression. The experiments were repeated three times.

### *In vivo* chemosensitivity assay

Six-week-old female BALB/c nude mice were used for the tumor formation assay. The mice were housed in an animal facility with a controlled environment, maintained at a temperature of 23 ± 2°C, 60 ± 5% humidity, under a 12:12 h light:dark cycle, with *ad libitum* access to sterilized food and water. A549 cells were transfected with pcDNA-GAS5 or empty vector and harvested from six-well culture plates after 48 h. Then, the cells were resuspended in a 50% RGF matrigel (BD Biosciences) solution in PBS at a concentration of 2 × 10^7^ cells/ml and injected in the right flank of the mice in a 100-μl volume. All of the mice were treated with gefitinib at 100 mg/Kg/day. The tumors were measured every 5 days using calipers. The tumors were resected from all of the mice after 30 days, and the subcutaneous growth of each tumor was calculated using the equation *V* = 0.5 × *D* × *d*^2^ (*V*, tumor volume; *D*, tumor length; *d*, tumor width). All of the procedures were performed under sodium pentobarbital anesthesia, and all measurements were acquired in a manner that minimizes suffering.

### Serological tumor marker analysis

The levels of serum CEA, CA19-9, and CA125 were measured using an Elecsys 2010 machine (Roche Diagnostics, Basel, Switzerland). The cutoff values were 4.3 ng/ ml, 27 U/ml, and 35U/ml for CEA, CA19-9, and CA125, respectively.

### Statistical analyses

All statistical analyses were performed using the SPSS version 18 software (Chicago, IL, USA). Two-tailed Student’s *t*-test, one-way ANOVA, and chi-square test were used to analyze the data. The values were expressed as the means ± standard deviations. Values of *P* ≤ 0.05 were considered statistically significant.

## Abbreviations

NSCLC: Non-small cell lung cancer
EGFR: Epidermal growth factor receptor
TKI: Tyrosine kinase inhibitor
LncRNA: Long non-coding RNA
GAS5: Growth arrest-specific 5
IGF-1R: Insulin-like growth factor 1 receptor
